# The Predictors of COMLEX-USA (Comprehensive Osteopathic Medical Licensing Examination of the United States) Level 1 Success Among Osteopathic Medical Students: The Role of Study Habits and Post-baccalaureate Background

**DOI:** 10.7759/cureus.94638

**Published:** 2025-10-15

**Authors:** Reagan Shults, Sabrina Belizaire, Christina Kennedy, Kim Chosie

**Affiliations:** 1 Medicine, Alabama College of Osteopathic Medicine, Dothan, USA; 2 Physiology, Alabama College of Osteopathic Medicine, Dothan, USA; 3 Medical Education, Alabama College of Osteopathic Medicine, Dothan, USA; 4 Research, Alabama College of Osteopathic Medicine, Dothan, USA; 5 Faculty of Student Success, Alabama College of Osteopathic Medicine, Dothan, USA

**Keywords:** board exam, comlex, comlex level 1, first-time pass rates, osteopathic medical student, postbaccalaureate program, question bank, study habits, truelearn, uworld

## Abstract

Commercial question banks (QBs) and post-baccalaureate (Post-Bac) pathways are widely used to prepare for COMLEX-USA (Comprehensive Osteopathic Medical Licensing Examination of the United States) Level 1, but their association with first-attempt pass rates in the pass/fail era is uncertain. We conducted a retrospective study at a single U.S. osteopathic medical school to examine whether (1) completion of UWorld and TrueLearn and (2) Post-Bac status are associated with first-attempt COMLEX Level 1 pass rates. Two analytic samples were used: a QB-engagement sample with usage data (n=541) and a Post-Bac comparison sample with status data (n=847). Exposures were the percentage of UWorld and TrueLearn completed before the exam and Post-Bac vs. non-Post-Bac educational background; the outcome was first-attempt pass (binary). Among passers vs. non-passers, the mean UWorld completion was 60.1% ±30.9 vs. 48.3% ±35.7 (Cohen’s d=0.38; small-to-moderate), and TrueLearn completion was 42.9% ±21.0 vs. 38.5% ±20.3 (d=0.21; small). Pass rates were 97.5% (199/204) for Post-Bac vs. 96.0% (616/643) for non-Post-Bac (risk ratio (RR)=1.02, 95% CI: 0.99-1.05; risk difference (RD)=1.75%, 95% CI: −0.88% to 4.38%). Across analyses, p-values did not reach statistical significance. In this cohort, neither QB completion nor Post-Bac status showed a statistically significant association with first-attempt pass; precision was limited by a few failures. Based on the findings, we recommend collecting more granular engagement metrics (e.g., timed vs. tutor mode, practice-test trajectories) and adjusting for academic confounders in future multi-site studies.

## Introduction

Preparation for COMLEX-USA (Comprehensive Osteopathic Medical Licensing Examination of the United States) Level 1 commonly involves commercial question banks (QBs) - vendor‑branded platforms such as UWorld and TrueLearn - used across both COMLEX and United States Medical Licensing Examination (USMLE) preparation. Prior literature links greater practice‑question volume, timed‑mode practice, and fewer, deeper resources with better licensing outcomes across settings, though findings vary by cohort and methodology [1-4]. In the pass/fail era, ceiling effects and reduced variance in outcomes likely attenuate measurable associations [5]. Systematic reviews/meta‑analyses synthesizing third‑party resource use in medical education further characterize this landscape [6-7]. This study aims to contribute to pass/fail-era evidence by reporting explicit effect sizes and confidence intervals for question-bank engagement and post-baccalaureate (Post-Bac) status in relation to first-attempt COMLEX Level 1 pass at a single institution.

We focus narrowly on two questions: (1) Is higher completion of UWorld and/or TrueLearn associated with first‑attempt COMLEX Level 1 pass? (2) Do Post‑Bac students differ in pass rates from Non‑Post‑Bac peers? We treat the study as exploratory and avoid causal claims. The pedagogic context is not measured here. Student learning strategies, structured guidance, and well‑being-related supports may influence outcomes but were not captured here; these are noted as context and future directions rather than exposures in the present analyses [8-9].

## Materials and methods

Study design and setting

We adopted a retrospective observational cohort study design using de‑identified academic data from the Alabama College of Osteopathic Medicine (ACOM). Data were collected before August 1, 2025. The dataset included students who matriculated in 2019, 2020, 2021, 2022, and 2023. For the present Level 1 analyses, first‑attempt outcome data were available through the Class of 2026 as of the cutoff date and were analyzed accordingly; matriculants without a recorded first‑attempt outcome by the cutoff (e.g., remediation/leave/attrition) were excluded from outcome analyses.

Ethical considerations

This project received approval through ACOM’s Institutional Review Board under Exempt Category 4 determinations (secondary research using identifiable private information collected for a purpose other than this study): IRB #25-05-07-001 - The impact of Post Graduate STEM Hours on First-time COMLEX I Pass rate. Approval date: 06/02/2025. IRB #25-05-06-001 - Retrospective analysis of ACOM students' percent complete of TrueLearn and UWorld question bank questions correlation to first-time COMLEX Level I pass rate. Approval date: 05/30/2025.

Records were accessed by institutional staff, then de‑identified before analysis for this manuscript. The IRB waived informed consent for this secondary analysis in accordance with the exemption.

Participants

Two analytic samples were defined a priori: (a) the QB engagement sample included students with available usage metrics for at least one vendor (n=541); (b) the Post‑Bac status sample included students with documented Post‑Bac status (n=847). Students lacking the relevant exposure data for each analysis were excluded from that analysis. Students without a recorded first‑attempt COMLEX Level 1 outcome by August 1, 2025 (e.g., remediation, leave of absence, attrition) were excluded from outcome analyses.

Data sources and measurement

Outcome

First‑time COMLEX Level 1 pass (binary).

Exposures

QB completion (UWorld; TrueLearn): Defined as the percentage of the vendor bank completed within a cohort’s subscription cycle corresponding to the level of training (ACOM provides vendor subscriptions aligned to OMS‑2 and OMS‑3 preparation). At the end of each subscription cycle (August/September), vendors report percentage complete to ACOM’s Medical Education Division; these reports were subsequently de‑identified and provided for research use. For the present Level 1 analysis, we used the completion metric corresponding to the OMS‑2 academic year for each cohort. Note: QBs at ACOM are vendor‑branded (UWorld, TrueLearn) rather than exam‑labeled; students may use them toward COMLEX and/or USMLE preparation.

Post‑Bac status from registrar/Student Affairs records: Coded Post‑Bac if a formal Post‑Bac program was completed before matriculation; otherwise Non‑Post‑Bac. Program‑level attributes (certificate vs. MS, duration, GPA thresholds) were not available.

Inclusion/exclusion criteria

We included matriculants from 2019-2023 with a recorded first-attempt Level 1 outcome by August 1, 2025. We excluded students missing vendor usage metrics from the QB analysis: none beyond undocumented status (if any) were excluded from the Post‑Bac analysis. We report counts of excluded students and reasons in Results.

Statistical analysis

Descriptive statistics were used to summarize exposures by outcome. Two‑group mean differences (pass vs. non‑pass) were compared using Welch’s t‑tests; we report Cohen’s d with 95% confidence intervals (CIs). Pass rate contrasts by Post‑Bac status were measured using chi‑square tests; we report risk ratio (RR) and absolute risk difference (RD) with 95% CIs. Given only a few (n=19) failures, multivariable adjustment was not attempted; we provide a post‑hoc detectable‑effect discussion. All tests are two‑sided (α=0.05). Analyses were exploratory; we emphasize estimates and precision over dichotomous significance.

## Results

Cohort and missing data

**Figure 1:**
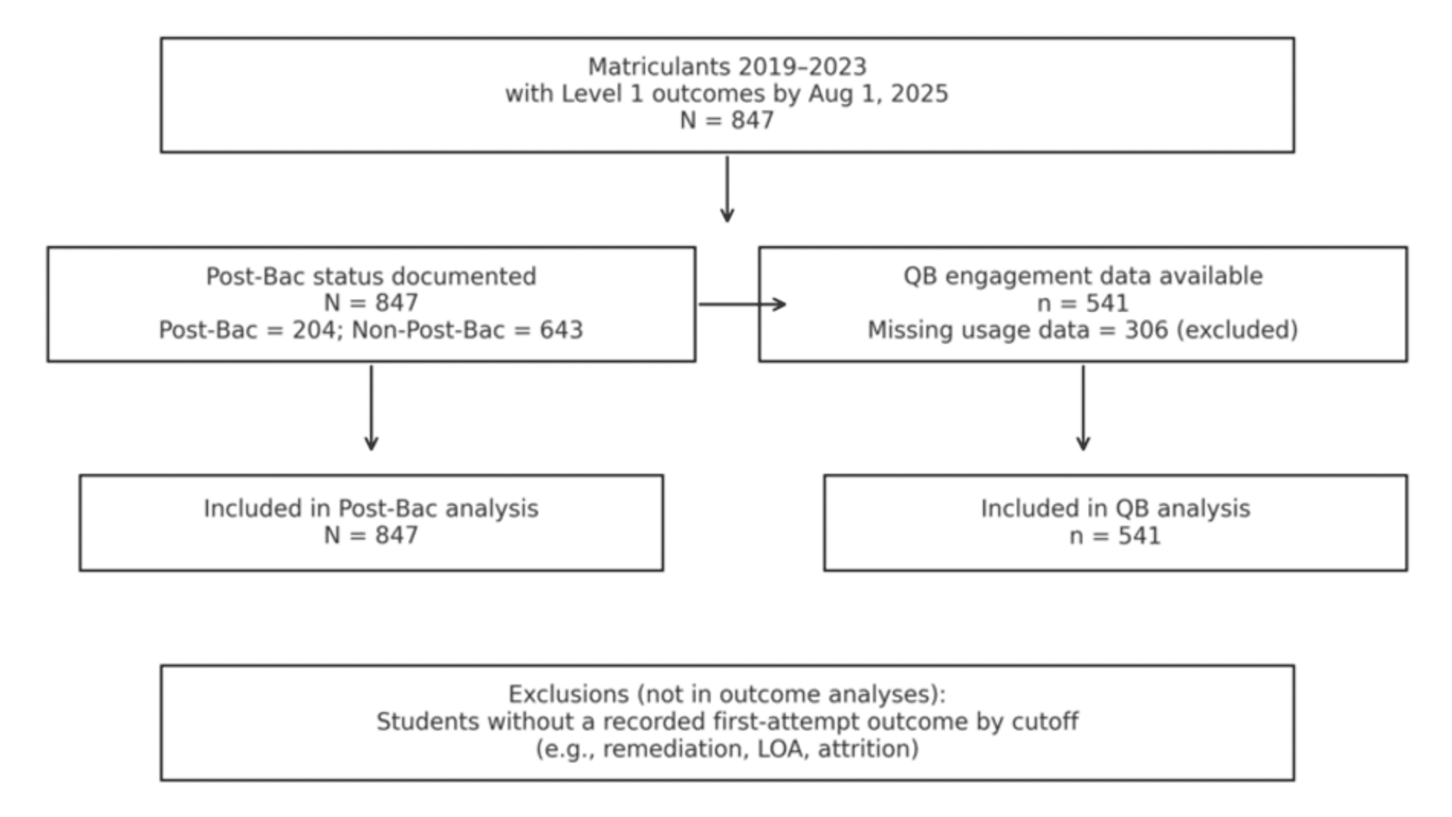
Cohort flow diagram Flow of students from the overall cohort (matriculants in 2019–2023 with Level 1 outcomes by Aug 1, 2025; n=847) into the two analytic samples. Post‑Bac status was available for 847 students (Post‑Bac: 204; non‑Post‑Bac: 643). QB engagement metrics were available for 541; 306 lacked vendor usage data and were excluded from the QB analysis. Students without a recorded first‑attempt outcome by the cutoff (e.g., remediation, LOA, attrition) were excluded from outcome analyses QB: question bank; LOA: leave of absence

Across matriculant classes during 2019-2023 with Level 1 outcomes recorded by August 1, 2025 (n=847), the first‑attempt pass was high; failures were rare (n=19, 2.2%). Usage metrics were available for 541 students; thus, 306 students lacked vendor usage data for analysis (typically due to unavailable exports or use outside the defined window). Students without a recorded first‑attempt outcome by the cutoff (e.g., remediation/leave/attrition) were excluded from outcome analyses. No imputation was performed. The cohort flow diagram is shown in Figure 1. Findings are summarized in Table 1.

**Table 1:** Analytic samples and missing data Counts are shown for matriculants in 2019–2023 with first‑attempt COMLEX Level 1 outcomes recorded by Aug 1, 2025 (n=847). “QB engagement data available” reflects vendor percent‑complete reports delivered to ACOM’s Medical Education Division at end‑of‑subscription (Aug/Sep) and subsequently de‑identified for research. Students without a recorded first‑attempt outcome by the cutoff (e.g., remediation, LOA, attrition) were excluded from outcome analyses. QB: question bank; LOA: leave of absence

Domain	Subset	Number of students	%	Notes
Overall analytic cohort	Matriculants 2019–2023 with Level 1 outcomes by Aug 1, 2025	847	100	Students without a recorded first‑attempt outcome by the cutoff were excluded from outcome analyses
Post‑Bac status documented	Yes	847	100	Sourced from registrar/Student Affairs records
	Post‑Bac	204	24.1	Program‑level attributes (certificate vs. MS, duration, GPA thresholds) not available
	Non‑Post‑Bac	643	75.9	—
QB engagement data available	Yes	541	63.9	Vendor percent‑complete reports at end‑of‑subscription (Aug/Sep); see Methods
	No (missing usage data)	306	36.1	Typically due to unavailable export or activity outside the reporting window

QB completion vs. pass (exploratory)

**Figure 2:**
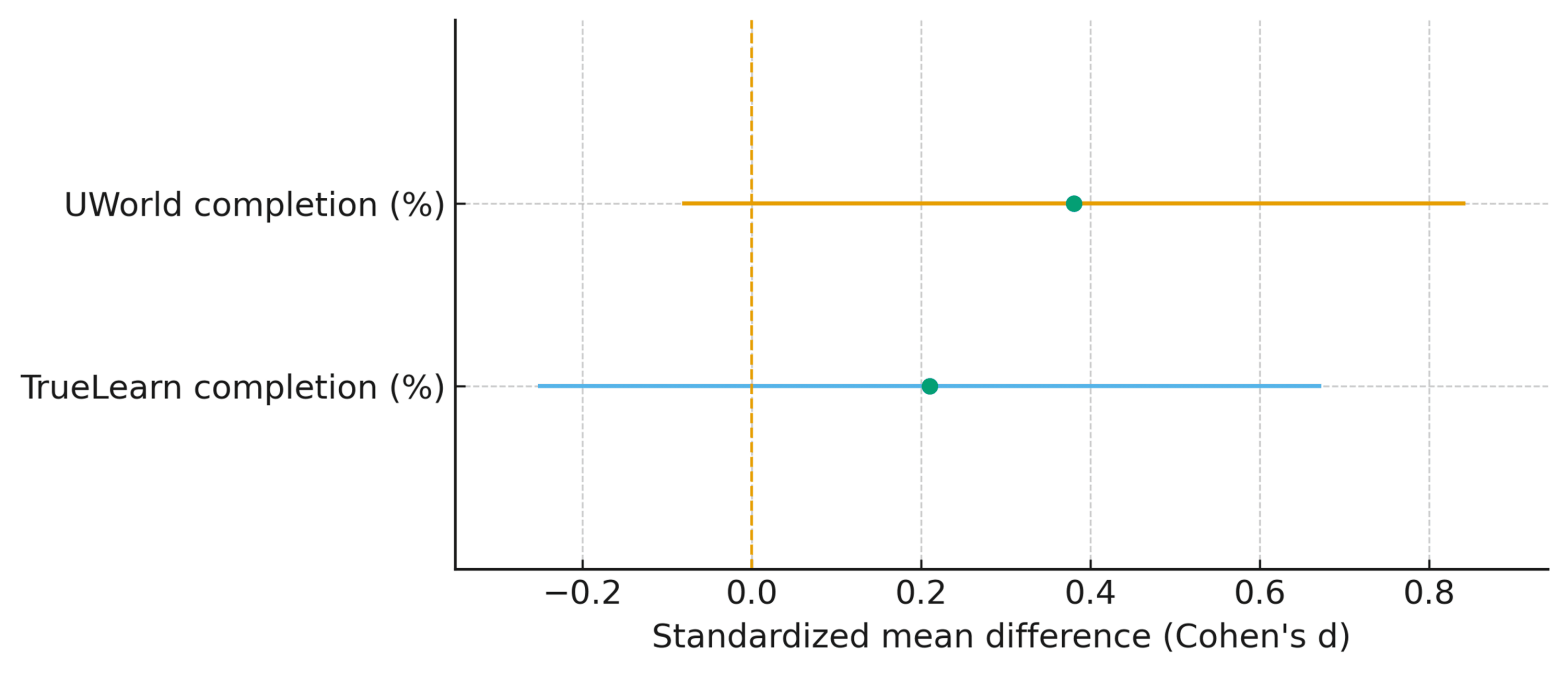
Forest plot of standardized mean differences Cohen’s d (with 95% CIs) for UWorld and TrueLearn completion comparing passers vs. non‑passers. The dashed vertical line denotes no difference (d=0). We did not overlay the Post‑Bac effect on this axis to avoid mixed scales CI: confidence interval

Among students with usage data (n=541), those who passed completed a greater proportion of UWorld than those who did not pass (60.1% ±30.9 vs. 48.3% ±35.7), corresponding to a small-to-moderate standardized mean difference (Cohen’s d=0.38, 95% CI: −0.08 to 0.84). This difference did not reach statistical significance (Welch t=1.42, df=18.99, p=0.171). For TrueLearn, passers likewise completed a higher percentage than non-passers (42.9% ±21.0 vs. 38.5% ±20.3), yielding a small effect size (Cohen’s d=0.21, 95% CI: −0.25 to 0.67) that was not statistically significant (Welch t=0.93, df=19.43, p=0.365). Effect estimates are summarized in Table 2 and displayed in Figure 2.

**Table 2:** Effect size summary for primary contrasts Cohen’s d/Hedges’ g quantify standardized mean differences between passers and non‑passers. Risk ratio (RR) and risk difference (RD) summarize Post‑Bac vs. non‑Post‑Bac first‑attempt pass rates. CIs are 95% two‑sided SD: standard deviation; CI: confidence interval

Comparison	Groups (n)	Estimate (mean ±SD or % [x/N])	Effect size and 95% CI	P-value
UWorld completion (%)	Pass (522) vs. non‑pass (19)	60.1 ±30.9 vs. 48.3 ±35.7	Cohen’s d=0.38 (95% CI: −0.08 to 0.84)	>0.05
TrueLearn completion (%)	Pass (522) vs. non‑pass (19)	42.9 ±21.0 vs. 38.5 ±20.3	Cohen’s d=0.21 (95% CI: −0.25 to 0.67)	>0.05
First‑attempt pass rate, Post‑Bac vs. non‑Post‑Bac	Post‑Bac: 97.5% (199/204) vs. non‑Post‑Bac: 96.0% (616/643)	—	RR=1.02 (95% CI: 0.99–1.05); RD=+1.75% (95% CI: −0.88% to +4.38%)	>0.05

Post‑Bac status vs. pass rate

The first-attempt pass rate was 97.5% (199/204) among Post-Bac students and 96.0% (616/643) among non-Post-Bac students. This corresponds to a risk ratio of 1.02 (95% CI: 0.99-1.05) and an absolute risk difference of +1.75% (95% CI: −0.88% to +4.38%). The between-group difference was not statistically significant (χ²=1.30, df=1, p=0.254).

Precision and power context

Estimates have wide CIs due to few failures and missing QB data. Detectable effect sizes at α=0.05 were larger than those observed; consequently, non‑significant results should be interpreted as inconclusive, not evidence of equivalence.

Full confidence intervals and exact test statistics are provided in the supplementary table in the Appendix.

## Discussion

Principal findings

In this single‑school, pass/fail‑era cohort, neither overall QB completion nor Post‑Bac status showed a statistically significant association with first‑attempt COMLEX Level 1 pass. Observed standardized mean differences for UWorld and TrueLearn completion were small, and the absolute pass‑rate difference between Post‑Bac and non‑Post‑Bac students was ~1.75% with wide confidence intervals.

Comparison with prior work

Work across USMLE/COMLEX contexts generally links greater question volume, timed‑mode practice, and longitudinal assessment use with higher licensing performance [1-4,8-9]. Divergence from those reports here may reflect (a) pass/fail‑era ceiling effects limiting detectable variance [5]; (b) unmeasured confounding such as MCAT, pre‑clinical GPA, and course performance; and (c) the quality and timing of QB engagement (e.g., timed vs. tutor mode, review depth), which we did not capture [6-7]. Our null findings, combined with small effect sizes, underscore the possibility that in a pass/fail context, broad completion percentages may be insufficiently granular to capture meaningful differences without richer usage metrics.

Practical implications

Institutions should avoid over‑interpreting raw completion percentages as stand‑alone predictors. More informative dashboards would integrate time‑stamped usage patterns, mode of practice, practice‑test trajectories, and academic baselines. Evidence‑based guidance that emphasizes retrieval practice, spacing, and learner well‑being may be more impactful than brand‑specific recommendations [6-7].

Post‑baccalaureate context

Although pass‑rate differences were not statistically significant here, prior studies suggest that structured Post‑Bac experiences can support readiness and long‑term success, particularly for students from non‑traditional backgrounds [10-13]. Future work should incorporate program descriptors (degree type, duration, GPA thresholds) to examine the heterogeneity of effect.

Limitations

This study has a few limitations: retrospective single‑site design; few failures yielding wide CIs; missing QB usage for ~36% of students; lack of granular engagement metrics (timed vs. tutor, time‑on‑task, review depth); no adjustment for academic confounders [14]; Post‑Bac program heterogeneity unmeasured; outcome limited to pass/fail. These constraints may reduce precision and generalizability.

## Conclusions

In this cohort, no statistically significant associations were observed between QB completion (UWorld, TrueLearn) or Post‑Bac status and first‑attempt COMLEX Level 1 pass. The small number of failures and limited engagement granularity constrain inference. Institutions should focus on richer, longitudinal learning analytics and prospective designs to clarify which study behaviors meaningfully relate to outcomes in the pass/fail era. Future multi-site studies with time-stamped engagement and academic covariates are needed to determine which study behaviors, if any, meaningfully affect first-attempt pass in the pass/fail era.

## Appendices

**Table 3:** Detailed estimates and test statistics for primary contrasts Welch’s t-tests were used for continuous contrasts due to unequal variances and highly unequal group sizes; Hedges’ g was reported with small-sample correction and 95% CIs. For binary outcomes, we report risk ratio (RR) with log‑scale Wald CI, absolute risk difference (RD) with Wald CI, and Pearson chi‑square test without continuity correction. All p‑values are two‑sided (α=0.05). SD: standard deviation; RR: risk ratio; RD: risk difference; CI: confidence interval; df: degrees of freedom

Comparison	Groups (n)	Group estimates (mean ±SD or % [x/N])	Test statistic (df)	Effect size and 95% CI	P-value	Notes
UWorld completion (%)	Pass (522) vs. non-pass (19)	60.1 ±30.9 vs. 48.3 ±35.7	Welch t=1.42 (df=18.99)	Hedges’ g=0.38 (95% CI: −0.08 to 0.84)	0.171	Two-sided tests; unequal variances accounted for (Welch)
TrueLearn completion (%)	Pass (522) vs. non-pass (19)	42.9 ±21.0 vs. 38.5 ±20.3	Welch t=0.93 (df=19.43)	Hedges’ g=0.21 (95% CI: −0.25 to 0.67)	0.365	Two-sided tests; unequal variances accounted for (Welch)
First-attempt pass, Post‑Bac vs. non‑Post‑Bac	Post‑Bac: 97.5% (199/204) vs. non‑Post‑Bac 96.0% (616/643)	—	Pearson χ²=1.30 (df=1)	RR=1.02 (95% CI: 0.99–1.05); RD=+1.75% (95% CI: −0.88% to +4.38%)	0.254	Counts: Post‑Bac pass/fail=199/5; non‑Post‑Bac=616/27
